# Study on the enlargement index of femtosecond laser-assisted capsulorhexis in 2–6-year-old patients with congenital cataract

**DOI:** 10.1186/s12886-021-02184-y

**Published:** 2021-12-23

**Authors:** Menglian Liao, Da Guo, Shan Liao, Wenwen Zhang, Ding Lin, Qiongyan Tang

**Affiliations:** 1 AIER School of Ophthalmology, Central South University, Changsha, 410015 Hunan China; 2 Changsha AIER Eye Hospital, No. 388, Furong Middle Road, Changsha, 410015 Hunan China

**Keywords:** Anterior capsulorhexis enlargement index, Capsulorhexis diameter, Congenital cataract, Femtosecond laser-assisted cataract surgery

## Abstract

**Background:**

To identify the capsule enlargement index after femtosecond laser-assisted anterior capsulorhexis in 2–6-year-old children who underwent congenital cataract surgery.

**Methods:**

In this prospective case series study, femtosecond laser-assisted anterior capsulorhexis was performed in patients with congenital cataract, aged 2–6 years. The actual achieved capsulorhexis diameters were measured with Digimizer version 4.2.6. Correlation coefficient (r) and multiple linear regression analysis were used to evaluate the variables that could potentially influence anterior capsulorhexis enlargement index (E).

**Results:**

This prospective study enrolled 28 eyes of 22 patients with congenital cataract. The mean age of the patients at surgery was 4.67 years ±1.54 (standard deviation [SD]). “E” of the 28 cases was 1.211 ± 0.039 (SD). Correlation analysis showed that “E” correlated significantly with the anterior chamber depth (ACD) (r = − 0.469, *p* = 0.021) and axial length (AL) (r = 0.452, *p* = 0.027). The following formula was developed by using multivariable linear regression analysis: Predicted E = 1.177–0.052 × ACD + 0.009 × AL, R^2^ = 0.346 (F = 4.396, *p* = 0.046).

**Conclusions:**

The anterior capsulorhexis enlargement index and its calculation formula could help to set up an accurate programmed capsulorhexis diameter for femtosecond laser-assisted congenital cataract surgery in children aged 2–6 years. Thus, an appropriate actual capsulorhexis diameter could be achieved.

## Background

Congenital cataract is a relatively rare disease with an estimated prevalence of 2.2/10000–13.6/10000 worldwide [1]; however, it is the primary cause of childhood blindness. Congenital cataract accounts for 12–39% of pediatric blindness cases in developing countries [2]. The period from birth till the age of 6 years is important for visual development in children [3]. Surgery should be performed during this period in patients with lens opacity, which leads to an unclear visual axis [4]. Continuous circular capsulorhexis (CCC) is the key and challenging step in cataract surgery, which influences the intraocular lens (IOL) position and postoperative refraction [5–7].

Femtosecond laser-assisted cataract surgery (FLACS), which facilitates precise circularity and shape during cataract surgery, can make CCC safer and easier to achieve [8, 9]. However, the presently achieved capsulorhexis size is definitely larger than that programmed for children. Therefore, to estimate the ideal CCC size, this study aimed to identify the laser capsulorhexis enlargement index and evaluate the factors affecting the index in 2–6-year-old children with congenital cataract. This work will help clinicians to set up an optimal programmed capsulorhexis diameter (PCD) and get accurate actual CCC size.

## Methods

This prospective, consecutive case series study was conducted in patients with congenital cataract aged 2–6 years, who underwent FLACS between August 2017 and July 2019. All interventions were performed at the Changsha AIER Eye Hospital, AIER School of Ophthalmology, Central South University, Changsha, China. The study was approved by Changsha Aier Eye Hospital Review Board. Informed consent of the surgical video recording and publication of images and findings in this study was obtained from all patients’ guardians and parents. The study protocol complied with the tenets of the Declaration of Helsinki.

The inclusion criteria were as follows: children with monocular or binocular congenital cataracts, femtosecond laser-assisted CCC with primary IOL implantation and posterior capsulotomy with anterior vitrectomy under general anesthesia, no history of ocular injury, no corneal pathology, no preoperative glaucoma, and no history of other surgeries. Patients with pupil diameter < 6 mm with full pharmacological mydriasis, intumescent white cataract and those with intraoperative capsule rupture were excluded.

### Surgical technique

FLACS was performed by a single experienced surgeon (QY Tang) in a sterile operating room. Using the femtosecond laser system (LenSx version 2.30; Alcon Laboratories, Inc., Fort Worth, Texas), an anterior capsulorhexis was programmed with 6 μJ energy and 300 μm capsule delta up and down to create a 4.2–4.8 mm incision based on the surgeon’s personal experience. A 2.2-mm two-plane corneoscleral limbus incision and a 1.0-mm single-plane clear cornea side incision were created using sterile knives. After hydrodissection, nucleus removal and cortical aspiration were performed. Thereafter, an IOL was accurately implanted into the capsular bag. Viscoelastic present in the capsular bag and anterior chamber was completely sucked out. Then, the paracentesis was enlarged to 2 mm and used for irrigation. A 3–4-mm posterior capsulotomy was performed with anterior 23-gauge vitrectomy via the main incision and the paracentesis. Finally, the main incision was hydrated, and the paracentesis was closed with a 10–0 absorbable polyglycolic acid suture. The whole operation was recorded with a high-definition camera, and the recorded video was used for the following analysis.

### Video review and Capsulorhexis size measurement

**Fig. 1:**
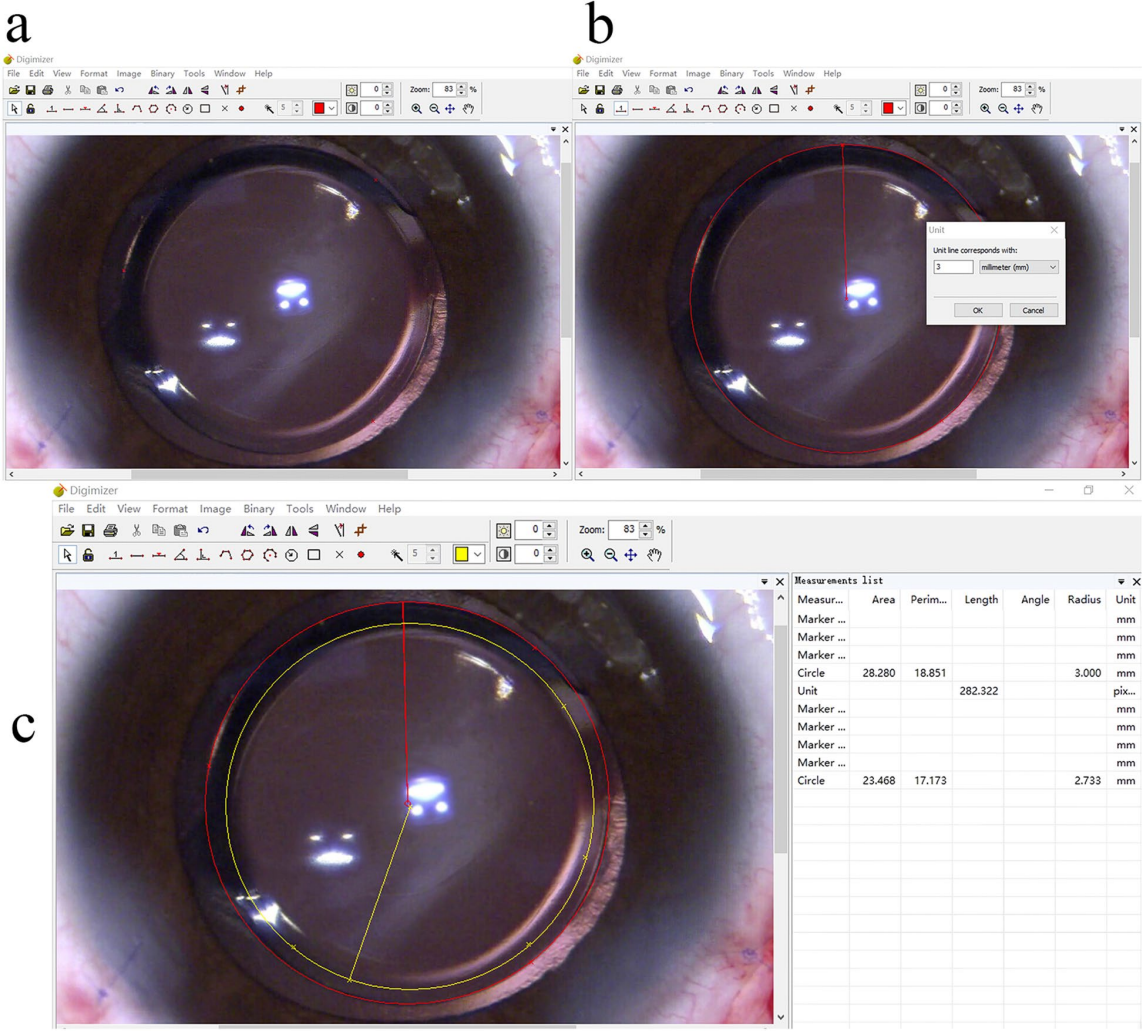
Measurement of the actual achieved capsulorhexis. **a** Select three points using the “marker style 1” (circled in blue). Delineate the boundary of the IOL optic using the “circle to center” (circled in red) to get the radius of IOL optic. **b** Click on the “Unit” button (circled in red). Use the radius of IOL optic to determine the unit. For example, if the diameter of IOL optic is 6.0 mm, type “3 mm” in the unit input box. **c** Delineate the boundary of the actual achieved capsulorhexis using the “circle to center” button to acquire the radius of capsulorhexis from the “measurement list” on the right side of the screen

Twenty-eight surgical videos of congenital cataract surgeries were reviewed. Images of the moment after suturing the incisions were captured and imported to a software for computer-assisted image analysis (Digimizer version 4.2.6, MedCalc Software Ltd., Mariakerke, Belgium). These images were used to measure the actual achieved capsulorhexis (Fig. 1). IOL is considered as the reference object to measure the actual achieved capsulorhexis diameter (AACD). Before performing the measurements, the radius of IOL optic is used to determine the unit. This measurement is crucial to assess the actual capsulorhexis. The detailed measurement procedure is shown in Fig. 1. The ratio of the AACD to the PCD was estimated as the capsule elasticity index (index of enlargement; E), which represents the degree of capsulorhexis enlargement.

### Clinical data collection

Before the cataract surgery, patients underwent a thorough ophthalmologic examination, including a slit lamp evaluation, intraocular pressure measurement, ophthalmoscopy after mydriasis, as well as biometry and keratometry measurement. Axial length (AL) and anterior chamber depth (ACD) measurements were obtained from IOL-Master500 (Carl Zeiss Meditec, Jena, Germany) and A-scan ultrasonography (Cinescan A/B, Quantel Medical, France). The keratometry values (K1 and K2) were measured using autorefractor keratometer RK-F1 (Canon Co, Tokyo, Japan). The above ophthalmic parameters, as well as the age at cataract surgery, patient sex, and PCD were collected in this study.

### Statistical analyses

Simple correlation analysis and partial correlation analysis were used to evaluate the factors contributing to capsulorhexis enlargement. The data of normal distribution (age at surgery, K1 and K2) were evaluated by Pearson’s correlation coefficient, while the non-normally distribution data (AL and ACD) were evaluated by Spearman’s rank correlation coefficient. A multivariable linear regression model with equation was used to identify the various factors that influences capsulorhexis enlargement. The correlation between right and left eyes was adjusted with Generalized estimating equation. All statistical analyses were performed using SPSS for Windows software (version 24.0, SPSS, Inc.), and a probability of < 5% (*p* < 0.05) was considered statistically significant.

## Results

Twenty-eight eyes of 22 patients (15 [68.18%] men and 7 [31.82%] women) who underwent FLACS with primary IOL implantation were included in this study. The mean age at surgery was 4.67 ± 1.54 years. All measurements were performed preoperatively. The ACD, AL, and IOL power of 6 cases were measured with A-scan ultrasound in children who could not cooperate with the IOL-Master500 examination by the same experienced technician [10].

Table 1 shows the characteristics of the eyes in the study population.

**Table 1:** Characteristics of patients’ eyes

Variables	Mean ± SD	Median	Range
Age at surgery (y)	4.67 ± 1.54	4.54	2.08, 6.92
AL (mm)	22.33 ± 1.71	22.12	20.14, 27.82
ACD (mm)	3.17 ± 0.51	3.33	1.57, 3.93
K1 (D)	42.36 ± 2.08	42.13	37.50, 46.37
K2 (D)	44.52 ± 1.67	44.31	41.98, 48.21
PCD (mm)	4.47 ± 0.13	4.40	4.20, 4.80

*K1* flat keratometry, *K2* steep keratometry, *ACD* anterior chamber depth, *AL* axial length, *PCD* programmed capsulorhexis diameter, *SD* standard deviation, *y* years

**Fig. 2:**
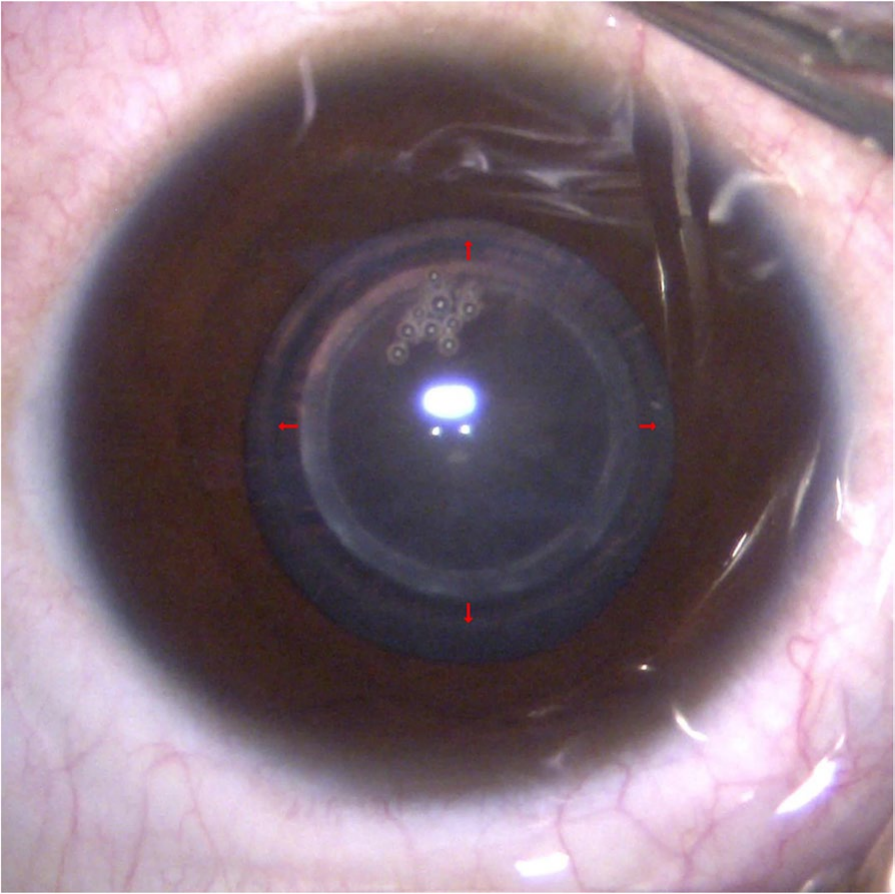
Symmetrical capsulorhexis enlargement immediately after femtosecond laser-assisted capsulorhexis in a child with congenital cataract

Anterior capsulorhexis was performed with femtosecond laser. No patient developed an anterior capsule tear. IOL implantation was successfully performed in all 28 cases. Tecnis ZCB00 (Abbott Medical Optics, United States), which is a hydrophobic acrylic 1-piece IOL with 6.0-mm optic diameter and 13.0-mm overall diameter, was implanted into the capsular bag. All the patients underwent implantation with the Tecnis ZCB00 IOLs. Posterior capsulotomy was successfully performed with anterior vitrectomy in all cases without any posterior capsule ruptures. Capsulorhexis enlargement occurred in all 28 cases (Fig. 2).

Table 2 shows the PCD, AACD, and “E” for each patient. “E” was estimated to be 1.211 ± 0.039 (standard deviation [SD]; range, 1.122–1.284).

**Table 2:** Clinical data of patients

Patient	Case	Eye	Age at Surgery (y)	Femtosecond Laser Capsulorhexis		
				PCD	AACD	E
Patient 1	1	L	6.83	4.5	5.48	1.218
Patient 2	2	L	4.50	4.5	5.47	1.216
Patient 3	3	R	6.00	4.6	5.57	1.211
Patient 4	4	L	5.00	4.6	5.55	1.207
Patient 5	5	R	6.75	4.8	5.72	1.192
Patient 6	6	R	3.50	4.6	5.64	1.226
	7	L	3.50	4.4	5.26	1.195
Patient 7	8	R	5.92	4.6	5.54	1.204
Patient 8	9	R	6.83	4.6	5.61	1.220
	10	L	6.83	4.6	5.59	1.215
Patient 9	11	R	3.25	4.6	5.67	1.233
	12	L	3.33	4.4	5.48	1.245
Patient 10	13	L	4.08	4.4	5.20	1.182
Patient 11	14	L	3.00	4.4	5.47	1.243
	15	R	3.00	4.4	5.46	1.241
Patient 12	16	L	2.08	4.5	5.78	1.284
Patient 13	17	L	6.92	4.5	5.45	1.211
Patient 14	18	R	4.83	4.4	5.17	1.175
Patient 15	19	R	5.50	4.4	5.14	1.168
Patient 16	20	R	2.75	4.4	5.45	1.239
Patient 17	21	R	4.33	4.4	4.95	1.125
	22	L	4.58	4.4	5.02	1.141
Patient 18	23	L	3.92	4.4	5.60	1.273
Patient 19	24	L	6.33	4.5	5.05	1.122
Patient 20	25	L	2.25	4.4	5.38	1.223
Patient 21	26	R	3.33	4.4	5.55	1.261
Patient 22	27	L	5.75	4.2	5.10	1.214
	28	R	5.83	4.2	5.17	1.231
Mean ± SD			4.67 ± 1.54	4.47 ± 0.13	5.41 ± 0.23	1.211 ± 0.039

*AACD* actual achieved capsulorhexis diameter, *PCD* programmed capsulorhexis diameter, *y* years, *L* left, *R* right, *E* index of enlargement ($\frac{\mathrm{actualachieved}\kern0.35em \mathrm{capsulorhexis}\ \mathrm{diameter}}{\mathrm{programmed}\ \mathrm{capsulorhexis}\ \mathrm{diameter}}$)

### Correlations among the different parameters and “E”

**Fig. 3:**
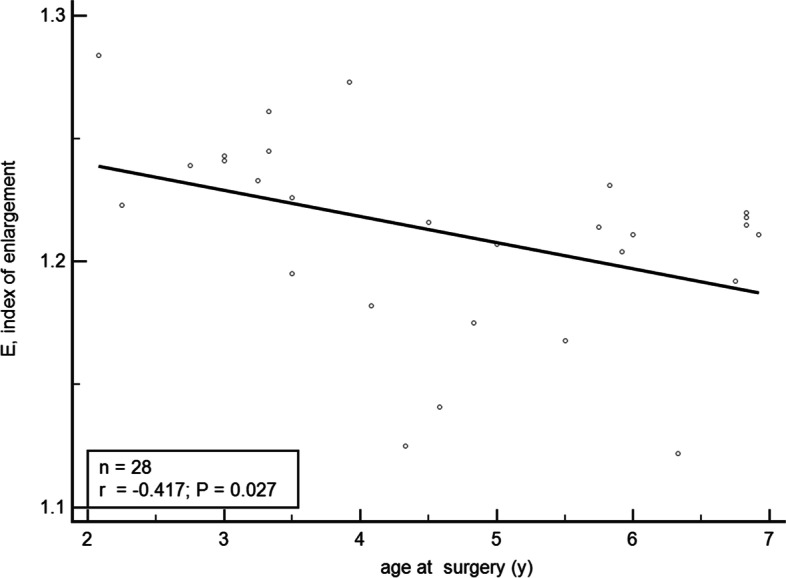
Relationship between “E” and the age at surgery. E, index of enlargement

**Fig. 4:**
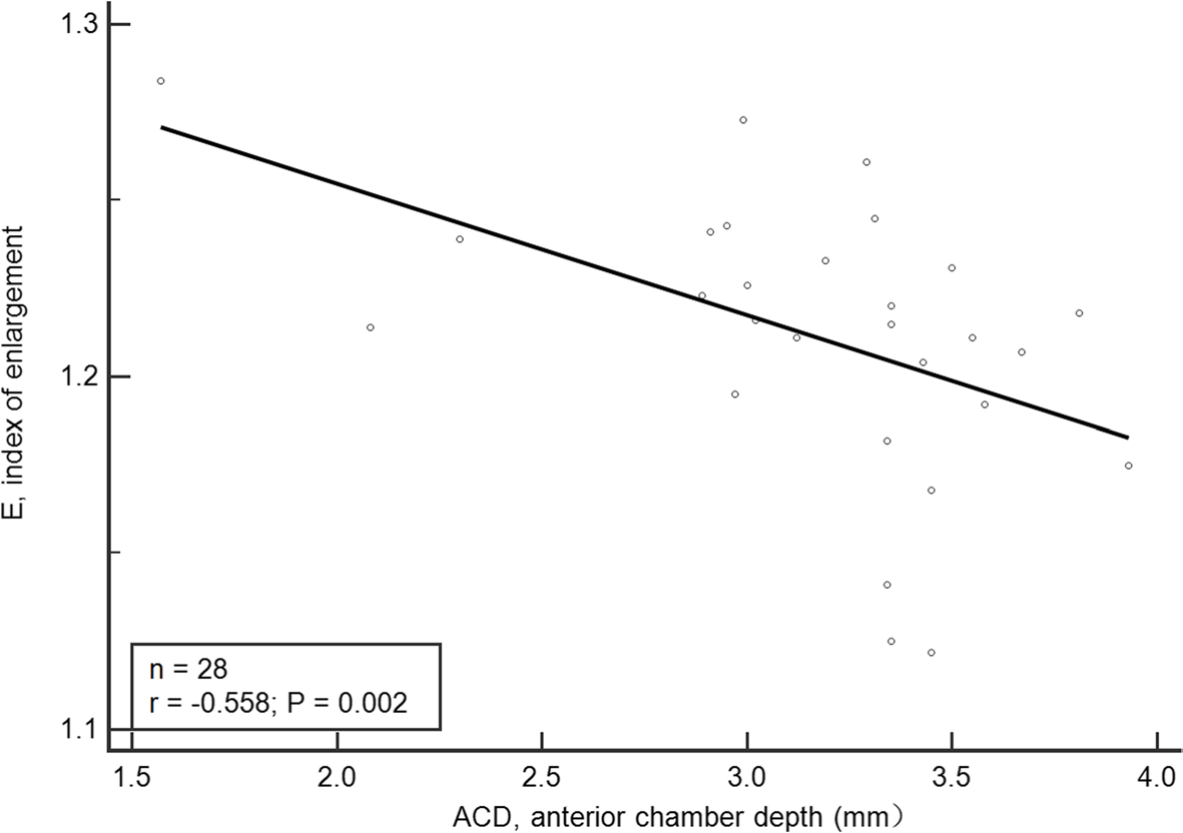
Relationship between “E” and ACD. E, index of enlargement; ACD, anterior chamber depth

Simple correlation analysis showed “E” was negatively related to the age at surgery (r = − 0.417, *p* = 0.027) (Fig. 3) and ACD (r = − 0.558, *p* = 0.002) (Fig. 4).

Results of partial correlation analysis showed that “E” value correlated significantly with ACD (r = − 0.469, *p* = 0.021) and AL (r = 0.452, p = 0.027), but showed only a weak correlation with the age at surgery (r = − 0.343, *p* = 0.100), K1 (r = − 0.253, *p* = 0.232) and K2 (r = 0.072, *p* = 0.737).

### Multiple linear regression

Multiple linear regression was performed using the age at surgery, AL, ACD, K1 and K2; the results showed that “E” correlated positively with AL, whereas it correlated negatively with ACD (Table 3).

**Table 3:** Multiple linear regression for “E”

Variables	Beta	SE	*p* value
ACD	−0.052	0.014	0.001
AL	0.009	0.004	0.046
Constant	1.177	0.083	< 0.001

*PCD* programmed capsulorhexis diameter, *ACD* anterior chamber depth, *AL* axial length, *SE* standard error

The multiple linear regression model was based on the following equation: *Predicted E = 1.177–0.052 × ACD + 0.009 × AL,* R^2^ = 0.346 (F = 4.396, *p* = 0.046). Furthermore, the predicted PCD formula (Eq. 1) was obtained from the “predicted E” equation

1 $$Predicted\ PCD=\frac{attempted\ capsulorheis\ diameter}{1.177-0.052\times ACD+0.009\times AL}$$

## Discussion

The age of primary IOL implantation in pediatric cataract surgery is an on-going debate, with the general consensus being 2 years old, and the feasibility of IOL implantation in children under 2 years old remains debatable [4, 11, 12]. In this study, 2–6-year-old children were included. To minimize the effects of some other surgical factors on anterior capsulorhexis, all 28 cases underwent the same surgical procedure: FLACS with primary IOL implantation and posterior capsulotomy with anterior vitrectomy. Posterior capsule capsulotomy was successfully performed in all cases to avoid PCO because young children may not comply with the Nd:YAG laser capsulotomy.

From 2009, femtosecond laser technology, which uses optical coherence tomography to perform real-time imaging of the anterior segment, has been successfully integrated into cataract surgery [13, 14]. Previous studies have reported that femtosecond laser-assisted CCC has excellent advantages in adults such as more precise, accurate, reproducible, and predictable outcomes [15, 16]. However, for children, high capsule elasticity generally results in an immediate significant increase in the capsulorhexis diameter after laser capsulorhexis [17–19]. In this study, anterior capsulorhexis enlargement occurred in all cases. Currently, when selecting the optimal PCD before femtosecond laser treatment, surgeons only can make judgments based on personal empirical rules. There is no clinically recommended value that can be used to avoid oversized opening of the anterior capsules; therefore, the actual achieved capsulotomy diameter is not always perfect.

A prospective study of 22 pediatric eyes (aged, 0.17–18 years) have reported that the age at surgery was the most important factor which influenced capsulorhexis enlargement, with younger children having a greater increase in the actual capsulotomy diameter than older children [20]. It constructed a formula to estimate the programmed anterior and posterior diameter related to age, but it has a small sample size and a large age range. This study analyzed the influence of some other factors (e.g. AL, ACD, K1, K2) on the capsulorhexis enlargement besides the age at surgery. Figure 3 shows the negative correlation of “E” value with the age at surgery. This result is consistent with the former research. But partial correlation analysis and multiple linear regression analysis have showed no significant correlation of “E” value with the age at surgery. Maybe it’s because we have narrowed down the age group. In the 2–6-year-old age group, the influence of age on the degree of capsulorhexis enlargement may not be considered. More parameters were included in this study. Figure 4 shows the negative correlation of “E” value with ACD. For the same PCD, a shallow preoperative ACD led to a large size of actual capsulorhexis diameter. ACD in the 28 cases examined in this study ranged from 1.57–3.93 mm. Of note, the intracapsular pressure and the anterior capsule tension may be higher in the eyes with shallower preoperative ACD. Moreover, the possible laxity of the lens zonular fibers may lead to greater changes in shallower ACD before and after surgery. A combination of the two factors maybe lead to more obvious enlargement of the capsulorhexis size after the femtosecond laser treatment. The AL of the 28 cases ranged from 20.14–27.82 mm. Data in Table 3 indicates that “E” correlated positively with AL. The authors speculate it may be due to the laxity of the lens zonular fibers also. However, the exact mechanism needs further study. Results of multiple regression analysis showed that “E” is closely related to AL and ACD. The predicted PCD formula (Eq. 1) is obtained based on the multiple linear equation of “Predicted E.”

The ideal CCC diameter should be slightly smaller than the optic diameter of the IOL [21]. The mean “E” value in this study was 1.211 ± 0.039 (SD) (range, 1.122–1.284). In clinical settings, according to the attempted capsulorhexis diameter, a PCD can be roughly calculated using the “E” value. Meanwhile, the predicted formula can provide clinicians with more accurate PCD values before the femtosecond laser procedure.

This study is limited by a small sample size. A larger sample size might show a more accurate consequence of prediction. To make the predicted PCD calculations convenient and faster, the following study will propose development of an automatic and intelligent software platform on which doctors only need to input ACD, AL, and attempted capsulorhexis diameter, and then the PCD could be generated immediately.

## Conclusions

The achieved capsulorhexis size is significantly larger than the programmed value for children after femtosecond laser-assisted anterior capsulorhexis. For the 2–6-year-old patients with congenital cataract, the “E” value and the predicted formula related to AL and ACD may help to set an optimal PCD to obtain a more accurate achieved capsulorhexis size.

## Data Availability

The datasets used and/or analyzed during the current study are available from the corresponding author on reasonable request.

## Abbreviations

CCC: Continuous circular capsulorhexis
IOL: Intraocular lens
FLACS: Femtosecond laser-assisted cataract surgery
E: Index of enlargement
AACD: Actual achieved capsulorhexis diameter
PCD: Programmed capsulorhexis diameter
ACD: Anterior chamber depth
AL: Axial length
K1: Flat keratometry
K2: Steep keratometry
